# Endocannabinoids in the Gut

**DOI:** 10.1089/can.2016.0001

**Published:** 2016-02-01

**Authors:** Nicholas V. DiPatrizio

**Affiliations:** Division of Biomedical Sciences, School of Medicine, University of California, Riverside, Riverside, California.

**Keywords:** cannabinoid receptor type 1, endocannabinoid, enteric nervous system, gut–brain, microbiome, peristalsis, vagus nerve

## Abstract

Cannabis has been used medicinally for centuries to treat a variety of disorders, including those associated with the gastrointestinal tract. The discovery of our bodies' own “cannabis-like molecules” and associated receptors and metabolic machinery—collectively called the endocannabinoid system—enabled investigations into the physiological relevance for the system and provided the field with evidence of a critical function for this endogenous signaling pathway in health and disease. Recent investigations yield insight into a significant participation for the endocannabinoid system in the normal physiology of gastrointestinal function and its possible dysfunction in gastrointestinal pathology. Many gaps, however, remain in our understanding of the precise neural and molecular mechanisms across tissue departments that are under the regulatory control of the endocannabinoid system. This review highlights research that reveals an important—and at times surprising—role for the endocannabinoid system in the control of a variety of gastrointestinal functions, including motility, gut–brain-mediated fat intake and hunger signaling, inflammation and gut permeability, and dynamic interactions with gut microbiota.

## Introduction

The endocannabinoid system is ubiquitously expressed throughout the rodent and human body and serves a multitude of physiological roles, including the regulation of gastrointestinal function.^1,2^ Activating cannabinoid receptors within the gut inhibits peristalsis and gastric acid secretion and enhances food intake.^1,3,4^

Evidence also suggests that dysregulation of the endocannabinoid system might play a role in intestinal disorders, including inflammatory bowel disease, irritable bowel syndrome, as well as obesity.^1,5^ For example, single-nucleotide polymorphisms in genes for constituents of the endocannabinoid system—including fatty acid amide hydrolase (FAAH), the degradative enzyme for the endocannabinoid, anandamide, and cannabinoid type 1 receptor (CB_1_R)—are associated with increased colonic transport and irritable bowel syndrome.^6,7^ Indeed, pharmacological treatment in humans with the general cannabinoid receptor agonist, dronabinol, decreased postprandial colonic motility, and the efficacy of this treatment was altered in subjects with gene variants of FAAH or CB_1_R.^8^

While this review is not formulated to be a comprehensive review of the field of gastrointestinal function in health and disease, it will provide highlights of studies describing a unique role for endocannabinoids in these processes.

## Endocannabinoid System and Gut Motility

More than a decade before the discovery of CB_1_R in the rat brain^9–11^ and its endogenous ligands (i.e., the endocannabinoids^12,13^), evidence from several laboratories suggested that cannabinoids are important regulators of contractility within the gastrointestinal tract.^14–16^ For example, oral administration of Δ^9^-tetrahydrocannabinol (THC)—the primary psychoactive component of cannabis—inhibited gastrointestinal motility in mice, as measured by the passage of charcoal through the intestine.^14^ Morphine also reduced motility, with greater potency than THC; however, only the actions of morphine—but not THC—were inhibited by the mixed μ-opioid receptor antagonist–κ-opioid agonist, nalorphine. This result highlights an important difference between the pharmacological effects of cannabinoids and opioids and strongly suggested entirely distinct receptor pathways for the two systems in modulating gastrointestinal motility and likely many other physiological processes.

### Cannabinoid modulation of cholinergic neurotransmission in the enteric nervous system

**FIG. 1.**
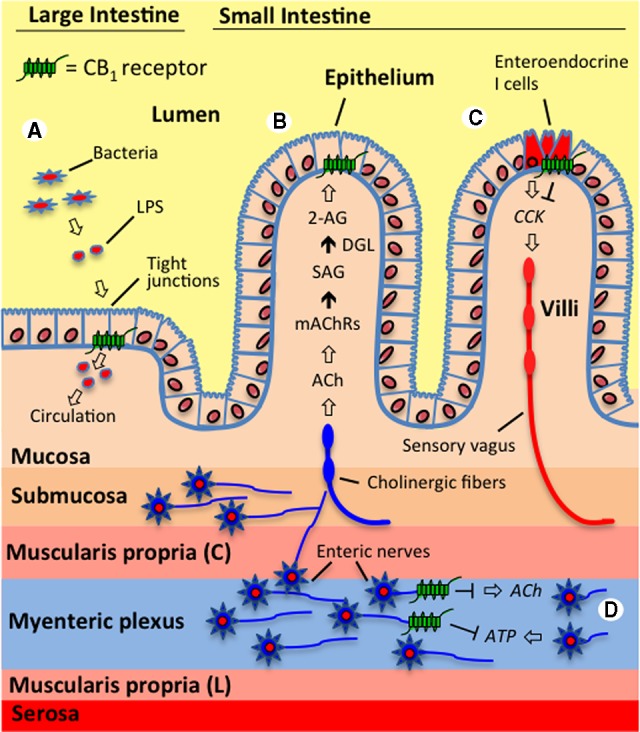
The endocannabinoid system controls a variety of gastrointestinal functions. **(A)** The endocannabinoid system in the large intestine is proposed to interact with gut microbiota and regulate epithelial barrier permeability. For example, activating cannabinoid type 1 receptors (CB_1_Rs) in mice increased circulating levels of lipopolysaccharide (LPS)—which is an endotoxin released from Gram-negative bacteria—through a proposed mechanism that includes decreased expression of the tight junction proteins, occludin and zonula occludens-1, and resulting increases in permeability.^76^ It is suggested that CB_1_Rs located in the intestinal epithelium control these processes. **(B)** Endocannabinoid signaling in the jejunum mucosa of the small intestine is triggered by fasting and tasting dietary fats and is proposed to be a general hunger signal that acts at local CB_1_Rs to inhibit satiation.^42,43^ The evidence suggests that during fasting, cholinergic signaling (acetylcholine, ACh)—possibly by the efferent vagus nerve—activates muscarinic acetylcholine receptors (mAChRs) in the small intestine, which, in turn, drive the conversion of the 2-arachidonoyl-*sn*-glycerol (2-AG) precursor, 1-stearoyl-2-arachidonoyl-*sn*-glycerol (SAG), into 2-AG through the activity of diacylglycerol lipase (DGL). Inhibiting subtype m_3_ mAChRs locally in the rat intestine blocked fasting-induced production of 2-AG in the jejunum mucosa and inhibited refeeding after a 24-h fast to the same levels as when a peripherally restricted CB_1_R antagonist was administered.^43^ **(C)** Endocannabinoid activity at CB_1_Rs located on small intestinal enteroendocrine I cells—which produce and secrete the peptide, cholecystokinin (CCK)—is suggested to promote feeding during a fast and drive the intake of fat-rich foods by inhibiting the release of CCK, which normally binds CCK receptors on the sensory vagus nerve and induces satiation after a meal.^42,43^ Supporting this hypothesis, the expression of CB_1_R mRNA on CCK-containing enteroendocrine I cells in the mouse small intestine has been reported,^59^ which suggests that CB_1_Rs in the gut mucosa control feeding by inhibiting the release of CCK and therefore indirectly modifying the activity of the sensory vagus. **(D)** Many studies provide evidence that CB_1_Rs on enteric nerves control intestinal contractility by inhibiting the release of the excitatory neurotransmitter, ACh.^1^ Recent studies also suggest that contractility is controlled by a dynamic interplay between the retrograde messengers, the endocannabinoids, and purines (e.g., adenosine triphosphate, ATP), which act in an opposing manner. It is proposed that the excitatory actions on contractility for ATP are mediated through increases in ACh, which are inhibited by the activation of prejunctional CB_1_Rs on enteric nerves.^33,34^ Both systems may functionally interact to regulate synaptic strength in the enteric nervous system.

Multiple lines of research began to elucidate the possible mechanism by which cannabinoids modulate intestinal transit. Studies from the 1970s showed that THC reversed cholinergic-mediated contractions within the guinea pig ileum, indicating that cannabinoids might act to reduce peristalsis via an—at the time—undiscovered receptor that inhibits the release of the excitatory neurotransmitter, acetylcholine, from enteric nerves in the gastrointestinal tract.^15–17^ Later *in vitro* work using human and rodent small intestinal tissues supported this hypothesis.^18–21^ Croci et al. reported that electrically evoked twitch responses in a human ileum longitudinal smooth muscle preparation were blocked by the general muscarinic acetylcholine receptor (mAChR) inhibitor, atropine, or the neurotoxin, tetrodotoxin (TTX), suggesting that twitch responses were mediated by cholinergic neurons.^19^ Importantly, application of the general cannabinoid receptor agonist, (+) WIN 55,212-2 (WIN), dose dependently inhibited twitch responses, and when WIN was applied in combination with atropine or TTX, no additive effects were observed. Furthermore, WIN was found to exert its effects in the ileum through activating CB_1_Rs because the selective CB_1_R antagonist/inverse agonist, rimonabant—but not the CB_2_-selective antagonist, SR144528—blocked WIN-mediated inhibition of twitch responses. These results suggest that CB_1_Rs control cholinergic neurotransmission in the human gastrointestinal tract and are the key regulators of contractility (Fig. 1D).

Several other research groups found similar results in the rodent small intestine, suggesting that interactions between endocannabinoid and cholinergic pathways in the gut are conserved across many species and likely serve a similar physiological role in the enteric nervous system to control gastrointestinal contractility.^18,20–22^ For example, Coutts and Pertwee reported that WIN and the nonselective cannabinoid receptor agonist, CP-55940, dose dependently inhibited twitch responses to electrical stimulation in the myenteric plexus longitudinal preparation of the guinea pig small intestine, an effect blocked by rimonabant.^18^ WIN or CP-55940 had no effect on contractility induced by exogenous application of acetylcholine, but rimonabant alone significantly increased the release of acetylcholine, indicating a prejunctional site of action for CB_1_Rs in controlling endogenous acetylcholine release. Studies by Izzo et al. found a similar effect on contractility in the guinea pig ileum circular smooth muscle for WIN and the endocannabinoid, anandamide.^20^ Both cannabinoid receptor agonists dose dependently inhibited cholinergic- and noncholinergic-mediated contractile responses to electrical field stimulation, and all effects were blocked by rimonabant. Importantly, when rimonabant was given alone, contractile responses were enhanced but failed to affect exogenously applied acetylcholine-induced contractions, further indicating that CB_1_Rs control contractility by inhibiting acetylcholine release from enteric neurons. In the mouse, anandamide or the selective CB_1_R agonist, arachidonoyl-2′-chloroethylamide (ACEA), reduced spontaneous contractility of ileum longitudinal muscle in a dose-dependent manner.^21^ The actions of anandamide or ACEA were inhibited by rimonabant, but not the selective CB_2_R antagonist, AM630, again implicating CB_1_Rs in the response. Indeed, CB_1_R immunoreactivity was found to be colocalized with choline acetyltransferase in the myenteric plexus of rat and guinea pig, further supporting the hypothesis that CB_1_Rs interact with cholinergic neurons to regulate contractility.^23^ In addition, monoacylglycerol lipase (MGL), the primary degradative enzyme for 2-arachidonoyl-*sn*-glycerol (2-AG),^24^ was found to be heavily expressed throughout the rat small intestine and colocalized with the enteric neural marker, PGP 9.5.^25^ Blocking the degradation of 2-AG by pharmacologically inhibiting the activity of MGL with URB602 reduced whole-gut transit, an effect completely absent in CB_1_ null mice, suggesting that endogenously produced 2-AG acting at CB_1_Rs controls gut contractility.

Recent studies by the Sharkey group (Bashashati et al.^26^) found a similar role for the biosynthetic enzyme for 2-AG, diacylglycerol lipase (DGL),^27^ in the control of intestinal transit and constipation. In these studies, CB_1_^−/−^, wild-type controls, and a mouse strain with a constipated phenotype, C3H/HeJ, were used. Immunoreactivity for DGLα in the ileum and colon myenteric plexus colocalized mostly with the vesicular acetylcholine transporter in nerve processes and punctate terminals surrounding enteric nerves. The highest expression of DGL was found in the stomach and colon compared to levels in the duodenum and ileum. Inhibiting the degradation of 2-AG with the MGL inhibitor, JZL-184, decreased whole-gut transit in wild-type controls, but not in CB_1_^−/−^ mice, and enhanced the inhibitory effects of systemically administered 2-AG on transit. Scopolamine, an anticholinergic that inhibits mAChRs, and loperamide, a general opioid receptor agonist, were used to reduce contractility *in vitro* in the mouse ileum and colon during electrical field stimulation, and the effects of inhibiting 2-AG biosynthesis by DGL with orlistat or OMDM-188 were evaluated. DGL inhibition reversed the inhibitory effects on contractility induced by scopolamine or loperamide. Similar results were found for orlistat or OMDM-188 on whole-gut transit when systemically administered to mice. Importantly, normalization of scopolamine or loperamide effects on transit was found in wild-type mice, but not in CB_1_^−/−^ mice, indicating that DGL likely exerts its actions on intestinal transit through the biosynthesis of 2-AG, which acts at local CB_1_Rs. C3H/HeJ mice had reduced fecal output when monitored for more than 1 h, and DGL inhibition with OMDM-188 increased output. Together, the results suggest that contractility and fecal output in the mouse ileum and colon may fall under the regulation of 2-AG signaling at local CB_1_Rs.

In contrast to the above work describing a role for CB_1_Rs—but not for CB_2_Rs—in the human and rodent small intestines on cholinergic-mediated contractility, pharmacological evidence suggests that both, CB_1_Rs and CB_2_Rs, control cholinergic neurotransmission in the mouse stomach.^28^ Both, atropine and TTX, abolished intraluminal pressure changes following electrical field stimulation to the mouse stomach, implicating cholinergic neurotransmission in the response. Interestingly, the effects of electrical field stimulation on intraluminal pressure were inhibited by all test compounds, including WIN, anandamide, and ACEA, as well as the selective CB_2_R agonists, JWH015 or JWH133. The evidence suggests that cholinergic-mediated gastric functions can be regulated through cannabinoid activity at both CB_1_Rs and CB_2_Rs differentially across specific organs in the gut (i.e., stomach vs. small intestine).

Boesmans et al. reported that CB_1_Rs might also control mitochondrial transport in enteric nerves.^29^ *In vitro* experiments using cultured guinea pig myenteric neurons revealed that spontaneous activity was increased in the presence of rimonabant and another selective CB_1_R antagonist, AM251, but inhibited when CB_1_Rs were activated with anandamide or methanandamide, a stable analogue of anandamide. Activity was blunted in the presence of URB957 and AA-5HT, both inhibitors of anandamide degradation by FAAH, highlighting the presence and role for endogenously synthesized CB receptor ligands in mediating enteric activity. Importantly, mitochondrial transport among enteric neurons was enhanced by the CB_1_R antagonist and decreased in the presence of the CB_1_R agonists, suggesting that CB_1_Rs might control energy metabolism in these cells. Further studies, however, will be necessary to confirm the precise physiological relevance of CB_1_Rs in mitochondrial function in the gastrointestinal tract.

Important studies also suggest that the endocannabinoid system in the gut is altered after consumption of high-fat diets, which is proposed to influence gut motility.^5,30^ Mice were fed a high-fat diet for 8 weeks, and small intestinal levels of anandamide and 2-AG were quantified, as well as intestinal transit.^5^ Interestingly, levels of anandamide in the small intestine were decreased, while levels of 2-AG increased, and these effects were met with increases in intestinal transit. Given the well-established evidence of a decrease in gastrointestinal motility following CB_1_R activation,^1^ that levels of 2-AG are on average 10–20 times higher than those for anandamide in the same tissue, and that 2-AG acts as a full agonist at CB_1_ compared to the partial agonist, anandamide,^31^ the results were surprising. The authors concluded that decreases in small intestinal content of anandamide led to the observed increases in transit.^5^ Endocannabinoids in these studies were extracted from whole homogenates of intestine, including mucosal and serosal layers, and thus make it difficult to ascertain whether specific areas within the intestine contain varying levels of the endocannabinoids, which could have distinct functional consequences on intestinal transit. An in-depth analysis of the subcellular localization for the endocannabinoid system as well as studies investigating the contribution of modified endocannabinoid signaling in obesity for gastrointestinal function are warranted.

### Interactions between purinergic and endocannabinoid systems

The purinergic endogenous signaling molecules, adenosine and adenosine triphosphate (ATP), are regulators of gastrointestinal contractility, acting primarily through P1 and P2 receptors, respectively.^32^ Recent evidence suggests that endocannabinoids and purines act in concert to regulate contractility, as well as synaptic strength and plasticity in the gastrointestinal tract. For example, Baldassano et al. investigated the interaction between these two pathways on spontaneous contractions in the mouse ileum.^33^ Activating CB_1_Rs with ACEA or P2 receptors with α,β-MeATP dose dependently inhibited spontaneous contractions, an effect blocked by atropine or TTX, confirming the established role for CB_1_Rs and P2 receptors in mediating cholinergic neurotransmission in the enteric nervous system. Importantly, the actions of ACEA on contractility were not modified by the P1 receptor antagonist, theophylline, but were completely inhibited by the P2 receptor antagonist, PPADS. The results provide substantial evidence of a functional interplay between CB_1_Rs and purinergic signaling at P2 receptors in cholinergic-mediated motor functions in the gastrointestinal tract (Fig. 1D).

Elegant studies by the Sharkey group and colleagues (Hons et al.^34^) suggested a further role for enteric endocannabinoid and purinergic interactions in a specific form of synaptic plasticity, known as metaplasticity (i.e., “plasticity of the plasticity”^35,36^). Immunohistochemistry revealed the colocalization of CB_1_Rs with the vesicular acetylcholine transporter and the synaptic marker, synaptotagmin, in the myenteric plexus of the mouse small intestine. Furthermore, interganglionic fibers in the myenteric plexus of CB_1_R-null (CB_1_^−/−^) and wild-type control mice were stimulated, and synaptic events were analyzed by intracellular microelectrodes placed in associated neurons. The results suggest that endocannabinoids act at presynaptic CB_1_Rs to control plasticity of cholinergic neurotransmission via an intricate interplay with ATP, which is similar to the endocannabinoids, is released retrograde. In contrast to the proposed inhibitory influence for endocannabinoids on excitatory cholinergic neurotransmission and contractility, however, ATP is excitatory and increases the release of acetylcholine, which, in turn, is proposed to drive the further production of the endocannabinoids and dampen excitation. Thus, both systems act in a dynamic and opposing manner and may functionally interact to regulate synaptic strength in the enteric nervous system (Fig. 1D). Given the complexity of this proposed phenomenon, further studies will be critical for a more complete understanding of the specific mechanisms and functional consequences for gastrointestinal physiology.

### Endocannabinoids and vagal parasympathetic control of motility

The brain and gut bidirectionally communicate via the vagus nerve to control a variety of physiological processes,^37^ and mounting evidence suggests a key role for CB_1_Rs in modulating the activity of vagal neurotransmission that influences gastrointestinal function.^1,38^ Systemic administration of THC in anesthetized rats led to a long-lasting reduction in pyloric contractility and intragastric pressure,^39^ and these effects were eliminated in rats that underwent surgical disruption of the vagus nerve or by inhibiting CB_1_Rs with rimonabant. The important contribution of CB_1_Rs located on the vagus nerve was highlighted by recent studies from the Elmquist group.^38^ Mice were generated that lacked CB_1_Rs in the nodose ganglion and dorsal motor nucleus of the vagus, which communicate neurotransmission of the afferent and efferent vagus nerve, respectively. Surprisingly, the mutant mice displayed a phenotype that included increases in gastrointestinal motility compared to controls but no changes in daily food intake, fasting-induced feeding, body weight, and energy expenditure. Together, the results suggest that CB_1_Rs located directly on the vagus nerve participate in the control of peristalsis but are dispensable for maintaining feeding and energy balance. Proposed roles for intestinal endocannabinoid signaling in feeding and indirect interactions with the vagus nerve will be discussed in subsequent sections (see “Endocannabinoid System and Gut–Brain Signaling”).

## Endocannabinoid System and Gut–Brain Signaling

Endocannabinoid signaling mechanisms in the gut have been proposed to participate in the control of food intake and energy balance via indirect actions with the vagus nerve,^3,4^ which bidirectionally communicates neurotransmission between the gut and brain.^37^ The first suggestion of a peripheral mechanism for endocannabinoid control of feeding was made by Gomez et al.^40^ and substantiated over the years by several other groups.^41–46^ Fasting was found to increase production of the endocannabinoid, anandamide, in the rat proximal small intestine.^40^ Systemic administration of WIN or anandamide increased feeding, while rimonabant inhibited feeding, and these effects were absent following chemical ablation of sensory afferents with capsaicin. Together, the results imply a peripheral mechanism for endocannabinoid signaling in feeding that is mediated through the vagus nerve.

### Endocannabinoids in the gut and fat taste

**FIG. 2.**
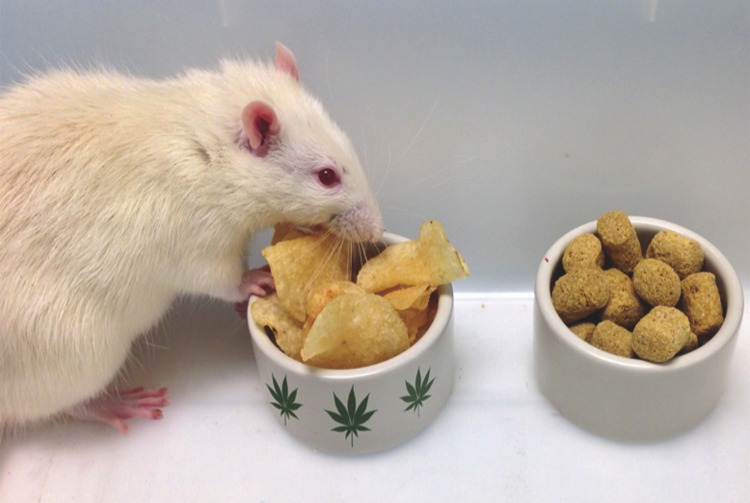
Fatty food intake is driven by gut–brain endocannabinoid signaling. Tasting dietary fat increases endocannabinoid levels within the rat jejunum.^42^ Inhibiting local endocannabinoid signaling at jejunal CB_1_Rs reduces fat intake and preferences for unsaturated dietary fats.^42,53^ Here, a rat prefers to eat fat-rich potato chips rather than a standard laboratory chow, which contains far lower quantities of dietary fat than chips. Thus, it is proposed for illustrative purposes that this rat's preference for the fat-rich food is driven by an enhancement of gut–brain endocannabinoid signaling (i.e., the body's natural “cannabis-like molecules”) that is triggered by tasting the fat contained in the chips.

Substantial evidence from numerous laboratories around the world suggests that mammals—including humans—display robust preferences for fatty foods based on their distinctive taste properties.^47–51^ In fact, in addition to the five established primary taste qualities (i.e., sweet, salty, bitter, sour, and umami), the notion of a fat taste has been recently accepted into the scientific lexicon as a sixth primary taste and given the name “oleogustus.”^52^ Several studies suggest that endocannabinoid signaling within the gut plays a key role in driving the intake of dietary fat due to its distinguishable taste properties.^3,4,42,48,53^ In these studies, a sham-feeding model in rats was used to isolate the taste component of feeding behavior from its postingestive consequences, and levels of the endocannabinoids, 2-AG and anandamide, were evaluated across tissue departments via liquid chromatography–mass spectrometry (see review for illustration and description of sham-feeding model^48^). Levels of 2-AG and anandamide doubled in the rat proximal small intestine (i.e., jejunum) after 30 min of oral exposure to dietary fat (i.e., a corn oil emulsion) compared to controls that were presented with an empty sipper tube.^42^ This effect was macronutrient specific, because carbohydrate or protein failed to produce changes in intestinal content of the endocannabinoids, and was organ specific, because no changes were found in all other organs tested (i.e., brain [dorsal striatum, ventral striatum, medial aspects of the hypothalamus, lateral aspects of the hypothalamus, parabrachial nucleus, and cerebellum], tongue, stomach, ileum, pancreas, and liver). Importantly, severing the vagus nerve completely blocked rises in jejunal endocannabinoid levels after tasting fat, suggesting that signals from the oral cavity in response to fat exposure are transmitted to the gut by vagal neurotransmission. Furthermore, inhibiting small intestinal CB_1_Rs with rimonabant or the peripherally restricted CB_1_R antagonist, URB447, blocked fat sham feeding, suggesting that this signaling event at jejunal CB_1_Rs is critical for the intake of dietary fats. Additional evidence for this hypothesis was provided by subsequent studies that evaluated the specific fatty acid requirements for driving jejunal endocannabinoid signaling.^53^ Sham-feeding emulsions containing the unsaturated free fatty acids, oleic acid (18:1 FFA) or linoleic acid (18:2 FFA), produced a similar increase in endocannabinoid levels in the jejunum as found for corn oil; however, tasting stearic acid (18:0 FFA), alpha-linolenic acid (18:3 FFA), or a nonnutritive oil (mineral oil) failed to elicit this response. In a two-bottle sham-feeding preference test, rats robustly preferred 18:2 FFA to mineral oil, and this effect was inhibited by the peripherally restricted neutral CB_1_R antagonists, AM6545 or URB447. Collectively, this series of studies provides evidence that palatable fatty foods containing unsaturated fats are consumed and preferred by rats due, in part, to the activation of endocannabinoid signaling at CB_1_Rs in the proximal small intestine, which signals to the brain to reinforce hedonic eating (i.e., nonhomeostatic feeding for pleasure^48^) (Fig. 2).

Similar to rats,^42,53^ recent studies provide evidence that hedonic eating by humans is associated with increases in endocannabinoid signaling. Circulating levels of the endocannabinoids were increased in normal-weight and obese humans during the anticipation of receiving, and after consumption of, a highly palatable food (i.e., chocolate) compared to a nonpalatable control diet.^54,55^ Furthermore, tasting a palatable sweet pudding was associated with significantly increased levels of circulating endocannabinoids in human subjects compared to levels after tasting a control or bitter pudding.^56^ Although the source of the circulating endocannabinoids is not known and extensive future experiments are required to determine specific mechanisms mediating the response, it is tempting to postulate that the endocannabinoids are synthesized in the small intestine in response to hedonic eating and are subsequently transported into circulation where they may influence central metabolic and reward pathways.

### Endocannabinoids in the gut as a general hunger signal

Building on the work above describing gut endocannabinoids and their proposed role in dietary fat taste and intake,^42,53^ recent evidence suggests a broader function for endocannabinoids in the mucosal layer of the small intestine, which may serve as a general hunger signal.^43^ Levels of 2-AG in the jejunum mucosa increased after fasting in a time-dependent manner compared to free-feeding rats, reaching significance by 24 h following the onset of food removal. Increases in levels of 2-AG were paralleled by elevations in the 2-AG precursor, 1-stearoyl-2-arachidonoyl-*sn*-glycerol (SAG), by 24 h after fasting (Fig. 1B). Both, SAG and 2-AG, rapidly returned to baseline free-feeding levels by 15 min after refeeding with chow. These data suggest a DGL-mediated conversion of SAG to 2-AG, which was confirmed by oral gavage of the DGL inhibitor, orlistat. Surprisingly, orlistat not only inhibited fasting-induced rises in jejunal 2-AG but also dramatically reduced levels far below those found during baseline free-feeding conditions. This result strongly suggests that DGL is the primary biosynthetic enzyme for 2-AG in the rat jejunum mucosa, although further investigations will be necessary to confirm this hypothesis. To identify the upstream mechanisms that participate in the production of jejunal 2-AG during fasting, an analysis of the role for cholinergic neurotransmission carried by the vagus nerve was evaluated. Studies conducted in the 1960s and 1970s found that (i) refeeding after a fast and (ii) sham feeding of a nutritional liquid diet by rats were blocked by the peripherally restricted general mAChR antagonist, atropine methyl nitrate, suggesting that cholinergic signaling in the periphery and an unknown downstream peripheral signaling mechanism are critical for maintaining feeding.^57,58^ Similar to results for sham-feeding corn oil,^42^ subdiaphragmatic vagotomy completely blocked fasting-induced rises in levels of 2-AG in the jejunum.^43^ Importantly, this blockade was mimicked by systemic administration of the general mAChR antagonist, atropine, or local intraduodenal administration of the selective m_3_ mAChR antagonist, DAU5884. Together, the results provide novel evidence suggesting that cholinergic neurotransmission—possibly carried by the efferent vagus nerve—activates jejunal m_3_ mAChRs, which, in turn, initiate the conversion of SAG to 2-AG through the DGL pathway (Fig. 1B). Further experiments revealed that this signaling event at CB_1_Rs in the jejunum might control feeding and serve as a hunger signal during times of energy depletion. Blocking peripheral CB_1_Rs with AM6545 inhibited refeeding after a 24-h fast, at a time when 2-AG activity in the jejunum is heightened. Similarly, inhibiting intestinal m_3_ mAChRs with an intraduodenal infusion of DAU5884—at a dose that effectively blocked fasting-induced rises in jejunal 2-AG—also significantly reduced refeeding. Importantly, when AM6545 and DAU5884 were coadministered just before refeeding, the quantity of chow consumed during refeeding was not reduced beyond levels found for each compound alone. Thus, it is proposed that cholinergic neurotransmission at jejunal mAChRs controls the production of 2-AG, which, in turn, activates local CB_1_Rs and serves as a general hunger signal (Fig. 1B, C).

Many gaps remain in our understanding of this complex interplay between the gut and brain in the control of feeding and energy balance, and ongoing studies are underway to address the specific upstream and downstream molecular and neural components that participate in the proposed gut–brain endocannabinoid hunger signaling. It has been suggested that endocannabinoids might act to increase feeding by *indirectly* modifying vagal signaling to the brain, which, in turn, provides a positive feedback that initiates the necessary motor functions to obtain food.^3,4^ Indeed, Sykaras et al. recently reported the expression of mRNA for CB_1_Rs in a subpopulation of enteroendocrine cells within the duodenum that are known to control meal size^59^ (Fig. 1C). Enteroendocrine I cells produce and secrete the peptide, cholecystokinin (CCK), which initiates satiation by binding CCK receptors located on the afferent vagus nerve.^60,61^ In turn, activation of CCK receptors on the vagus is thought to increase the firing rate of vagal inputs to the brainstem and terminate a meal. This indirect action for endocannabinoids on vagal activity is in line with results in mice, discussed in previous sections, whereby genetic depletion of CB_1_Rs located in the afferent and efferent vagus failed to modify food intake but affected gut motility.^38^ It is plausible that during times of energy depletion (e.g., fasting and starvation) when endocannabinoid activity in the gut is greatly elevated, the activation of CB_1_Rs on enteroendocrine I cells might inhibit the release of CCK, which, in turn, decreases afferent vagal neurotransmission and delays the onset of satiation. From a prosurvival perspective during times of feast or famine, this notion makes sense: Consume as many calories as possible because a next meal is never guaranteed. In modern times of readily accessible foods, however, this hunger signal might promote compulsive eating and obesity.^3^ Further studies, of course, are necessary to confirm this hypothesis and include (i) more selective vagotomies to determine the specific role for efferent vagal neurotransmission supplying the upper small intestine in endocannabinoid production, (ii) a systematic evaluation of the specific cell subtypes that contain the constituents of endocannabinoid system in the lumen of the jejunum and their proximity to each other, (iii) downstream pathways that signal to the brain to influence food intake, and (iv) extensive behavioral testing to ascertain the specific role that gut–brain endocannabinoid signaling serves in hunger and food reward.

## Endocannabinoids and Gastric Secretions

Substantial evidence suggests that the endocannabinoid system in the gastrointestinal tract controls gastric secretions. *In vivo* studies in mice conducted by Izzo et al. revealed that activating CB receptors with systemically administered WIN inhibited intraluminal fluid accumulation, gastrointestinal transit, and defecation.^62^ These effects were normalized by coadministration with rimonabant and increased when rimonabant was given alone, suggesting that endogenous activity at intestinal CB_1_Rs provides an inhibitory tone on these processes.^63^ *In vitro* experiments in the rat ileum found a similar inhibitory effect of CB_1_Rs on gastric secretions.^64^ For these experiments, an electrical current was applied to the ileum, stimulating gastric acid secretion. This response was reduced by WIN and reversed by coadministration with rimonabant, implicating CB_1_Rs in the actions of WIN.

Adami et al. reported that gastric acid secretions in the stomach also fall under the control of local CB_1_Rs.^65–67^ A lumen-perfused experimental design was used in anesthetized rats, which includes continuous perfusion of the stomach with saline through a cannula inserted into the esophagus, and perfusate is subsequently collected via a second catheter inserted into the duodenum to assess levels of gastric secretions. Intravenous administration of WIN or the potent mixed CB_1_–CB_2_ receptor agonist, HU-210, dose dependently inhibited secretions induced by pentagastrin or 2-deoxy-d-glucose. These effects were blocked by rimonabant but unaffected by the CB_2_R antagonist, SR144528, or the selective CB_2_R agonist, when given alone, highlighting the role for CB_1_Rs in response to CB receptor agonists. Bilateral cervical vagotomy blocked the inhibitory effects of HU-210 on gastric secretions, suggesting an important role for the vagus nerve in this response. Atropine, however, failed to affect responses to CB_1_R activation, suggesting that muscarinic receptors—which are targets for cholinergic neurotransmission in the gut^37^—were not involved. A more in-depth analysis of the specific neural and molecular mechanisms involved in CB_1_R-mediated regulation of gastric secretions is essential.

## Endocannabinoids in Inflammatory Bowel Disease

CB_1_Rs or CB_2_Rs are proposed to serve a protective role in inflammatory bowel disease, and a plethora of studies support the possible value of targeting these pathways with pharmacological agents for therapeutic gain.^1^ For example, oil of mustard (OM) administration into the colon of mice, which induces a severe colitis, was used to investigate the effects of stimulating CB_1_Rs and CB_2_Rs on inflammation associated with this model.^68^ Treatment with the selective CB_1_R agonist, ACEA, and the selective CB_2_R agonist, JWH133, both greatly reduced parameters of OM-induced colitis, including inflammatory damage and diarrhea. The specific role for CB_2_Rs in colitis was further examined by Storr et al. using a trinitrobenzene sulfonic acid (TNBS) model of colitis in wild-type and CB_2_^−/−^ mice.^69^ TNBS-induced colitis was inhibited by treatment with JWH133 and another selective CB_2_R agonist, AM1241. These effects were blocked by pretreatment with the CB_2_R antagonist, AM630, and exacerbated by treatment with AM630 alone. All CB_2_R ligands were ineffective in CB_2_^−/−^ mice. Furthermore, enhancing endogenous levels of anandamide by blocking its degradation by FAAH with URB597 or the endocannabinoid uptake inhibitor, VDM11, significantly reduced inflammation induced by TNBS.^70^ All effects of pharmacological treatments were absent in CB_1_^−/−^ or CB_2_^−/−^ mice. In addition, other studies using the TNBS model of colitis reported protective effects against inflammatory damage in mice lacking FAAH.^71^ Collectively, the results highlight the role for CB_1_Rs and CB_2_Rs in experimental models of colitis and provide strong evidence for the therapeutic value of cannabinoid-based therapeutics for the treatment of inflammatory bowel disease.

Inflammation in the gastrointestinal tract is associated with increased epithelial permeability due, in part, to a dysfunction of normal intestinal epithelial barrier function, and mounting evidence suggests a role for the endocannabinoid system in these processes.^1,72^ Alhamoruni et al. used an *in vitro* model for assaying epithelial permeability, whereby Caco-2 cell monolayers were treated with the cytokines, TNFγ and TNFα, to induce an inflammatory response and increase permeability.^72^ Surprisingly, THC or cannabidiol—both derivatives from the cannabis plant—ameliorated the effects of cytokine treatment on permeability, whereas anandamide or 2-AG enhanced permeability. Further suggesting a detrimental role for the endocannabinoids on epithelial permeability, inhibiting the activity of the enzymes responsible for anandamide or 2-AG breakdown with URB957 or JZL184, respectively, also increased permeability induced by cytokine treatment. Differential effects of exogenous versus endogenous cannabinoids on epithelial barrier functions in an experimental model of inflammation suggest a complex role for cannabinoid mechanisms in these processes and warrant further investigation across experimental models. Nonetheless, the results suggest a role for the endocannabinoid system in inflammation-induced disruptions in intestinal permeability and might be a target for therapeutics aimed at improving epithelial barrier function in disease.

## Endocannabinoids and the Gut Microbiome

Several recent studies suggest the possible interactions between the endocannabinoid system and gut bacteria, known as the microbiota.^73–75^ For example, 4-week treatment with the cannabinoid receptor agonist, HU-210, in lean mice significantly increased plasma levels of lipopolysaccharide (LPS),^76^ which is an endotoxin released from Gram-negative bacteria.^77^ Conversely, treatment with rimonabant for 12 days in obese *ob/ob* mice with gut barrier disruption and resulting metabolic endotoxemia (i.e., chronic increases in circulating LPS^78^) were met with reduced levels of plasma LPS and changes in the localization of the tight junction proteins, occludin and zonula occludens-1.^76^ This presumed improvement in endothelial barrier permeability for inhibition of CB_1_Rs was further indicated by *in vitro* experiments using a Caco-2 cell monolayer.^76^ Treatment with HU-210 enhanced LPS-induced decreases in the expression of mRNA for occludin and zonula occludens-1. Importantly, rimonabant—but not the CB_2_R antagonist, SR144528—completely inhibited changes in mRNA expression for both proteins and normalized transepithelial resistance after treatment with LPS and HU-210. Changes in the expression of mRNA for CB_1_R in the colon were also found in mice treated with probiotics, in germ-free mice compared to conventionally raised controls, and after antibiotic treatment or chronic access to a high-fat diet. In addition, treatment with probiotics induced changes in the expression of mRNA for biosynthetic and degradative enzymes of anandamide in white adipose tissue that were paired with decreases in adiposity, suggesting modifications to endocannabinoid-mediated adipogenesis. Collectively, these studies underscore the ability for CB_1_R activation to control endothelial barrier integrity and provide novel evidence for interactions between the endocannabinoid system, gut microbiota, and possibly adiposity. Furthermore, disorders that enhance intestinal endocannabinoid tone, including diet-induced obesity,^5^ could further exacerbate barrier function and pathology associated with metabolic endotoxemia, which itself has been shown to initiate the onset of diabetes and obesity.^78,79^ It will be important to further define the specific localization of CB_1_Rs within the epithelium for the reported effects on barrier function for microbiota–CB_1_R interactions and to define the role for local immune cells in these processes. Research into elucidating the complex interplay between host microbiota and the endocannabinoid system in normal health and disease is only in its infancy, but these recent experiments suggest an exciting area of future study.

## Abbreviations Used

2-AG: 2-arachidonoyl-*sn*-glycerol
ACEA: arachidonoyl-2′-chloroethylamide
Ach: acetylcholine
ATP: adenosine triphosphate
CB_1_R: cannabinoid type 1 receptor
CCK: cholecystokinin
DGL: diacylglycerol lipase
FAAH: fatty acid amide hydrolase
FFA: free fatty acid
LPS: lipopolysaccharide
mAChR: muscarinic acetylcholine receptor
MGL: monoacylglycerol lipase
OM: oil of mustard
SAG: 1-stearoyl-2-arachidonoyl-*sn*-glycerol
THC: tetrahydrocannabinol
TNBS: trinitrobenzene sulfonic acid
TNF: tumor necrosis factor
TTX: tetrodotoxin
